# Supplementation of Micronutrient Selenium in Metabolic Diseases: Its Role as an Antioxidant

**DOI:** 10.1155/2017/7478523

**Published:** 2017-12-26

**Authors:** Ning Wang, Hor-Yue Tan, Sha Li, Yu Xu, Wei Guo, Yibin Feng

**Affiliations:** School of Chinese Medicine, The University of Hong Kong, Pokfulam, Hong Kong

## Abstract

Selenium is an essential mineral naturally found in soil, water, and some of the food. As an antioxidant, it is one of the necessary trace elements in human body and has been suggested as a dietary supplement for health benefit. Although the human body only needs a trace amount of selenium every day, plenty of recent studies have revealed that selenium is indispensable for maintaining normal functions of metabolism. In this study, we reviewed the antioxidant role of nutritional supplementation of selenium in the management of major chronic metabolic disorders, including hyperlipidaemia, hyperglycaemia, and hyperphenylalaninemia. Clinical significance of selenium deficiency in chronic metabolic diseases was elaborated, while clinical and experimental observations of dietary supplementation of selenium in treating chronic metabolic diseases, such as diabetes, arteriosclerosis, and phenylketonuria, were summarized. Toxicity and recommended dose of selenium were discussed. The mechanism of action was also proposed via inspecting the interaction of molecular networks and predicting target protein such as xanthine dehydrogenase in various diseases. Future direction in studying the role of selenium in metabolic disorders was also highlighted. In conclusion, highlighting the beneficial role of selenium in this review would advance our knowledge of the dietary management of chronic metabolic diseases.

## 1. Introduction

With its name derived from the Greek word “Selene,” selenium has caught attention as a micronutrient since 1817, when it was first described as a by-product from sulphuric acid production. Although selenium is an essential element which is naturally occurring in the body, its endogenous level fluctuates across populations in different geographical areas, as well as different age groups in the same area, indicating that both environmental and internal factors may affect the selenium level [1, 2]. Both organic and inorganic forms of selenium can be absorbed by the small intestine and in turn can be widely distributed in various body tissues and render important biological functions, primarily through regulating the synthesis of selenoproteins [3]. Human selenoproteins are a series of 25 selenium-containing proteins whose synthesis requires insertion of a selenium-containing homolog of cysteine. The major role of multiple selenoproteins, such as glutathione peroxidase (GPX), thioredoxin reductase (TrxR), and iodothyronine deiodinases (IDD), is to act as important intracellular antioxidants in preventing oxidative injury [4]. Therefore, the importance of selenium supplementation in boosting up the internal antioxidative defence has been highlighted in recent years.

It was not until 1957 that the therapeutic role of selenium as a micronutrient was identified by Wrobel et al., who observed that selenium supplementation at a low dose can prevent a rat liver from necrosis [3]. Since then, mounting studies have suggested the beneficial effects of selenium supplementation in maintaining immune-endocrine function, metabolic cycling, and cellular homeostasis. In addition to its essential physiological function, the potential of selenium supplementation in remitting human pathological conditions, especially chronic metabolic disorders, has been frequently proposed. Wei et al. found that daily selenium intake has a negative correlation with metabolic syndromes [5]; however, the role of selenium supplementation as antioxidants in major metabolic syndromes, such as hyperlipidaemia and hyperglycaemia, has not yet been critically reviewed. Here, we retrieved studies from PubMed database and systematically reviewed the biological activity and underlying mechanism of selenium in various metabolic diseases. Toxicity and recommended dose of selenium were reviewed and discussed. In addition, as the molecular action of selenium was less identified, we predicted and discussed the potential interaction on gene networks and signalling proteins upon selenium supplementation.

## 2. The Role of Selenium in Treatment of Hyperlipidaemia

Hyperlipidaemia refers to a phenomenon of abnormal high concentrations of lipid products and lipoproteins in the blood. It could be primarily caused by the genetic and familial factors, but in most of the cases, it is triggered by other metabolic disorders. Secondary hyperlipidaemia is a kind of metabolic abnormality involved in several chronic human diseases, such as diabetes and obesity. Healthy young subjects with higher dietary selenium intake (higher than 82.4 *μ*g/day) showed lower level of sialic acid and triacylglycerol, and they exhibited reduced inflammatory response and prevalence of metabolic syndromes such as lipid profile impairment and insulin resistance [6]. It was found that increased hair selenium concentration in hyperlipidemic patients had adverse association with their lipid profiles [7]. Karita et al. showed that the selenium level in erythrocytes may be an indicative factor of decreased total cholesterol (TC) and low-density lipoprotein cholesterol (LDL-C) after menopause in Japanese premenopausal and postmenopausal women [8]. This indicated the beneficial effect of selenium intake in regulating lipid metabolism. In contrast, another study found that plasma selenium level was raised in preaging cases (aged 59–71) of lipemia [9].

In rats with hyperlipidaemia caused by diazinon, one of the most organophosphate insecticides used in agriculture and industry, selenium supplementation in the form of sodium selenite (200 *μ*g/kg/d) could normalize the serum thiobarbituric acid reactive substances (TBARS), total lipids, cholesterol, urea, and creatinine, which may be due to the induced antioxidant enzymes and glutathione content [10]. Additionally, nicotine reduced the intestinal intake of selenium and caused hyperlipidaemia in rats. Selenium supplementation (1 *μ*g/kg/d) improved the hyperlipidaemic condition, as evidenced by the reduced expression of hydroxymethylglutaryl-CoA reductase (HMGCoA) and lipogenic enzymes [11]. In Triton WR-1339-induced hyperlipidaemia, supplementation of selenium in the form of diphenyl diselenide (10 mg/kg) increased the high-density lipoprotein cholesterol (HDL-C) while reduced the non-HDL and triglyceride in the serum of mice, indicating its hypolipidemic effect [12]. But another study suggested that this effect was independent to its antioxidant property [13]. Furthermore, it was found that hyperlipidaemia had a significant adverse effect on male fertility, while supplementation of inorganic selenium or selenium-enriched probiotics (equivalent to 0.05 *μ*g/g Se) was suggested to improve fertility in humans and animals [14].

### 2.1. Arteriosclerosis

Kalkan et al. found that dyslipidemic patients with glycogen storage disease type I and type III, which did not lead to premature atherosclerosis, exhibited lower plasma concentration of selenium compared with healthy control [15]. Chan et al. found that selenium deficiency may be associated with reduced arterial function in patients, with higher potential of vascular incidents [16]. Supplementation of selenium in the form of selenium yeast (0.1 mg/kg) subsidized the cardiac enzymes, lipid peroxidation, and inflammation, indicating that it can improve myocardial performance by preventing oxidative damage [17]. Treatment of a formula containing selenium (10 ppm for 30 days) might modulate the lipid profile of hyperlipidaemic rats, mainly reducing the level of TC, non-HDL-C, and atherogenic index [13]. In contrast, another study in British adults showed that higher level of selenium in serum indicated an adverse cardiometabolic risk, with increased total and non-HCL cholesterol [18].

Supplementation of selenium was also suggested when antiatherogenic mode of nutrition was applied to patients, according to a study of 800 persons, in which the results indicated that sodium selenite treatment could give out a favourable outcome on the immune system [19]. Delattre and colleagues showed that treatment of LDL apheresis might be the direct cause of low plasma selenium in normocholesterolemic subjects [20]. This was further evidenced by the observation that LDL apheresis treatment, which eliminated cholesterol-containing LDL from bloodstream, could lower plasma level of selenium but not the other antioxidants including vitamin E and *β*-carotene [21]. However, an argument was raised on a long-term benefit of LDL apheresis treatment in reducing atherogenic cholesterol oxidation products (COP) in the plasma, and therefore acute drop of selenium by the treatment seemed not meaningful [22].

### 2.2. Hypercholesterolemia

The significance of serum selenium concentration was highlighted by the study from Galicka-Latala and colleagues. The lipid peroxidation marker malondialdehyde (MDA) had a negative correlation with serum selenium level in both normo- (plasma total cholesterol less than 5.2 mmol/L) and hypercholesterolemic (plasma total cholesterol greater than 5.2 mmol/L) patients, while the MDA, to be specific in low-density lipoprotein, was negatively associated with selenium level in patients diagnosed with hypercholesterolemia [23]. In an experimental model, deficiency of selenium in hypercholesterolemic animal led to lower expression of hepatic LDL receptor and HMG-CoA reductase but elevated apolipoprotein B (ApoB) level, which can be subsidized by selenium resupplementation (1 ppm) [24–26]. In high-fat diet-fed rats, treatment of selenite (0.173 mg/kg/d via gavage for 10 weeks) could suppress LDL-C in serum, triglyceride, and TC in the liver, which was probably due to the reduced expression of fatty acid synthase [27]. Kaur et al. found that supplementation of selenium (1 ppm) could diminish the high-fat diet-induced ROS levels by 29% and suppress the serum paraoxonase 1 but not platelet-activating factor acetylhydrolase, indicating its potential in limiting the complications of hypercholesterolemia [28]. Furthermore, selenium supplementation (1 ppm) could restore the reduced T3 and T4 hormones in the serum of high-fat diet-fed rabbits, with improvement of type I iodothyronine 5′-deiodinase (5′-DI) in the liver, indicating that selenium is capable of regulating thyroid behaviours in hyperlipidaemic state [29, 30]. Selenium supplementation (2.5 mg/kg, i.p.) was also found to improve dysregulated renal morphology caused by hypercholesterolemia [31].

## 3. The Role of Selenium in Treatment of Hyperglycaemia

A lot of studies have revealed that hyperglycaemic patients exhibited selenium deficiency in the blood, though a study in diabetic Germans showed that blood selenium level was higher in patients with hyperglycaemia [32]. Compared with some contradicting outcomes in the therapeutic effect of selenium supplementation on type 2 diabetes mellitus, it is quite a consensus that selenium is beneficial for patients with type 1 diabetes mellitus as well as for treatment of hyperglycaemia-related complications.

### 3.1. Type 1 Diabetes Mellitus

It was found that selenium was distinctly decreased in the red blood cells of type 1 diabetic patients and was negatively correlated with the elastic and viscous component of whole blood viscosity, indicating the selenium deficiency in red blood cells may be associated with impaired haemorpheology of type 1 diabetic patients [33]. Another study showed that selenium level in erythrocyte was lower in type I diabetic groups [34]. Sheng et al. treated alloxan-induced diabetic mice with sodium selenite (via gavage, 2 mg/kg/d for 4 weeks) and found that selenite reduced blood glucose and improved glutathione (GSH) levels in the liver and brain of diabetic mice; nonetheless, selenite treatment in normal mice surprisingly reduced hepatic GSH level [35]. Using STZ-induced diabetic model, Guney and colleagues found that combination of vitamin E (60 mg/kg/d) and sodium selenite (1 mg/kg/d) treatment decreased blood glucose level by inducing expression and activities of several antioxidant enzymes, such as catalase, superoxide dismutase, and GPX [36]. Similar antioxidant treatment could also reverse the skin lipid peroxidation and subsequent damage [37]. Furthermore, Satyanarayana et al. found that half or single therapeutic dose of selenium (0.9 and 1.8 *μ*g/200 g, resp.) had hypoglycaemic effect in alloxan-induced diabetic animal, while double dose of selenium (3.6 *μ*g/200 g rat) increased blood glucose. Combination treatment of selenium improved the hypoglycaemic effect of gliclazide in both normal and diabetic animals [38]. Atalay et al. compared the effect of oral administration of sodium selenate (0.3 mg/kg/d) and doxycycline on STZ-induced hyperglycaemic rats and concluded that selenate can reduce blood glucose level without triggering significant loss of body weight. Selenate preserved thioredoxin-1 (TRX-1) level in skeletal muscle but not in the liver, while the protein carbonyl capacity and oxygen radical absorbance capacity in the liver were suppressed. In addition, free and total protein thiol levels were restored by selenate treatment (0.3 mg/kg, p.o.) in both the skeletal muscle and liver of diabetic rats [39]. Bajpai et al. had similar conclusion about the hypoglycaemic effect of sodium selenite, another inorganic form of selenium in STZ-induced diabetic rats. Treatment of selenite (10–30 *μ*g/ml for 14 days) can reduce serum glucose and improve the wound closure of diabetic mice by normalizing the low levels of vascular endothelial growth factor (VEGF) and extracellular superoxide dismutase. It also improved angiogenesis in the wound site of diabetic rats [40]. Mechanistically, Chen et al. suggested that selenium (1 ppm) might play an insulin-like role to normalize the glucose metabolism and improve glucose uptake and metabolism in the liver of alloxan-induced diabetic animals [41]. Selenium supplementation (5 ppm/d for 4 weeks) could restore glucagon-like peptide 1 receptor (GLP-1R) expression and suppress insulin receptor substrate-1 (IRS-1) and Raf-1 in the liver, which may render hypoglycaemic effect on STZ-induced diabetic rats [42]. In addition, Kahya et al. showed that 1.5 mg/kg/d of sodium selenite treatment can improve brain and erythrocyte lipid peroxidation and plasma IL-1*β* and IL-4 levels due to the restoration of antioxidant status in STZ-induced diabetic rats [43]. Erbayraktar et al. compared the hypoglycaemic effect of different forms of selenium in STZ-induced diabetic rats and found that both sodium selenate and selenomethionine (2 *μ*mol/kg/day via orogastric route for 12 weeks) can suppress elevation of blood glucose in diabetic mice. However, sodium selenate seemed to have a stronger effect in inducing GPX activity than selenomethionine [44]. Xu et al. examined the combination effect of low-dose insulin and selenium (180 *μ*g/kg/d) in treatment of STZ-induced hyperglycaemia and found that this combination could facilitate reduction of blood glucose and lipid levels, with remarkable restoration of PI3K and GLUT4 in cardiac muscle, which eventually improved myocardial function [45]. Selenium supplementation (0.3 mg/kg Se) in the form of selenium-enriched *Catathelasma ventricosum* mycelia can normalize serum glucose, insulin, and antioxidant enzyme activity in STZ-induced diabetic mice and suppress *α*-amylase and *α*-glucosidase activities in *in vitro* gastric and intestinal models [46]. Supplementation of sodium selenite (intraperitoneal injection of 0.3 mg/d for 25 days) can increase vitamin E level in the liver and plasma of STZ-induced diabetic animals. Treatment of selenium can increase GPX activity and GSH concentration in the red blood cells and liver, which reduces TBARS concentration [47].

### 3.2. Type II Diabetes Mellitus

Anderson et al. found that in patients with type 2 diabetes the selenium level and antioxidant status in plasma remained normal, though 30% of the subjects may have Zn deficiency [48]. A clinical study conducted by Stranges et al. showed that selenium uptake (200 *u*g/d) had no significant beneficial effect to the incidence of type 2 diabetes. Nonetheless, in the highest tertile of baseline plasma selenium level, selenium statistically increased the risk for type 2 diabetes occurrence (hazard ratio, 2.70 (CI, 1.30 to 5.61)) [49]. Another study revealed that inactivation of selenium-dependent enzymes by glycation might eventually lead to oxidative stress in patients with type II diabetes [50]. Study on growing rats with developing obesity and diabetes, from Mueller and colleagues, revealed that a recommended dietary level or superanutritional level of selenium uptake (1-2 mg/kg in diet), in the forms of either selenite or selenate in diets, increased the body weight of rats. The expression of GPX1 in the liver was upregulated by selenium supplementation, which then triggered overexpression of PTP1B and reduction of glutathionylation [51]. Wang et al. reported that overexpression of GPX1 may deliver a beneficial effect by changing pancreatic expressions of PDX1 and UCP2 via elimination of ROS and hyperacetylation of H3 and H4 histone in islet. However, in long term, it may lead to chronic hyperinsulinaemia by dysregulating beta cell mass and pancreatic content [52]. Surprisingly, Zhou et al. found that, instead of being an antioxidant, selenium might foster lipid peroxidation and decrease GSH/GSSG in the liver and promote ASK1/MKK4/JNK oxidative stress pathway [53]. These observations revealed a plausible mechanism underlying the action of selenium supplementation on the development of obesity and diabetes [51]. Furthermore, Faghihi et al. observed, in a clinical study of type 2 diabetes patients, that selenium intake (200 *μ*g/d for 3 months) accelerated disease progression by increasing fasting plasma glucose, glycosylated haemoglobin A1c, and serum HDL-C level, indicating an unflavoured outcome of selenium uptake in type 2 diabetes despite the restoration of serum selenium level towards optimal concentration of antioxidant activity [54]. In contrast, an experimental observation in high-fat diet/STZ-induced type 2 diabetic rats showed that supplementation of selenium (180–500 *μ*g/kg/d) can reduce blood glucose, cholesterol, and triglyceride level and improve antioxidant status and nitric oxide (NO) release [55]. Additionally, treatment of selenium-containing tea polysaccharides (Se-GTP, 200–800 mg/kg/d for 8 weeks) in high fructose-induced resistant animals could significantly improve hyperglycaemia and hyperinsulinemia and restore antioxidant and hepatic lipid levels. However, this does not prove the direct effect of selenium supplementation in improving type 2 diabetic condition as no comparative study has been made to understand the independent efficacy of tea polysaccharides without selenium [56]. Similar concern was raised by the research from Tanko et al., which showed selenium-enriched yeast (0.1–0.2 mg/kg/d via oral administration for 6 weeks) can improve cholesterol diet-induced type 2 diabetes mellitus in rats by reducing blood glucose and increasing antioxidant activities, yet it could not rule out the possibility of independent therapeutic effect of nonselenium components in the yeast [57].

### 3.3. Gestational Diabetes

Al-Saleh et al. measured the serum concentration of selenium in gestational diabetic patients, and the results showed that plasma selenium was significantly lower (102.3 versus 75.2 *μ*g/L) [58]. Hawkes observed that pregnant women at between 12 and 34 weeks of gestation had a lower level of serum selenium, which was inversely correlated with increased fasting glucose, but not the insulin level, suggesting that selenium may affect glucose metabolism independent to insulin [59]. Bo and colleagues found that dietary intakes of selenium but not vitamins were significantly lower in hyperglycaemic subjects; in particular, the intake of selenium was negatively correlated with gestational hyperglycaemia. Selenium level was particularly lower in patients with impaired glucose tolerance [60]. However, maternal intake of selenium (6.3/95 *μ*g/d, mean/maximum) had neither positive nor negative correlation with the incidence of advance beta cell autoimmunity in early childhood [61]. Guney et al. applied a combination treatment of vitamin E (60 mg/kg/d) and sodium selenite (1 mg/kg/d) onto diabetic pregnant rats and found that after 21 days of treatment, the abnormal lipid peroxidation (LPO) level in rats was significantly normalized, which may be related to the potent increase of antioxidant enzymes [62]. Asemi et al. conducted a RCT clinical study of selenium supplementation in patients with gestational diabetes. The results indicated that selenium (200 *μ*g/d for 6 wk from weeks 24 to 28 of gestation) could significantly reduce fasting plasma glucose, serum insulin level, and insulin resistance. In addition, selenium could reduce serum high-sensitivity C-reactive protein and increase GSH, resulting in reduction of plasma MDA. However, there was no significant changes on *β*-cell function, lipid profiles, plasma NO, or total antioxidant capacity concentrations observed [63].

### 3.4. Hyperglycaemic Complications

The direct evidence of antioxidant effects of selenium in STZ-induced diabetes was obtained by Naziroglu and colleagues. Treatment of sodium selenite (0.3 mg/d for 21 days) improved vitamin E concentration, reduced MDA level in the plasma, and suppressed testicular lipid peroxidation, indicating that selenium supplementation may reduce reactive oxygen substances and improve testicular complications in diabetes [64]. Aliciguzel et al. found that in diabetic rats fed with 10% sucrose following alloxan injection, GPX activity was lower in the liver, brain, kidney, and heart in both early and late stages of diabetes [65]. Furthermore, Liu and colleagues found that supplementation of selenium in the form of Se-polysaccharide from *Catathelasma ventricosum* (100 mg/kg/d) could also reduce MDA and LDL-C in diabetic mice, which was associated with the increased antioxidant enzymes in the liver and kidney. These together with restoration of LDL-C rendered protective effect on the pancreas, liver, and kidney against peroxidative damage [66]. In addition, nanoparticles of selenium exhibited a beneficial effect (0.1 mg/kg via oral administration for 28 days) in improving the testicular tissue condition in STZ-induced diabetic rats. This was related to reduce lipid peroxidation and NO with increased glutathione content and antioxidant enzyme activities. Molecular studies showed that mRNA level of Bcl-2 was upregulated in testicular tissue of selenium nanoparticle-treated rats while Bax was suppressed. Treatment of selenium nanoparticles (0.1 mg of SeNPs/kg) increased PCNA expression as well as testicular function [67].

Faure et al. found that selenoprotein GPX activity in diabetic patients was lower than that in healthy subjects, which was associated with thrombosis and cardiovascular complications [68]. In STZ-induced diabetic animals, Ayaz et al. observed that sodium selenite treatment (i.p. 5 *μ*mol/kg/d for 4 weeks) could prevent myofibril loss and reduce myocyte size. Selenium supplementation (5 *μ*mol/kg/d) rendered remission on discus intercalaris and nucleus in the heart and preserved myofilament and Z-lines [69]. Treatment of sodium selenite (10 *μ*mol/kg/d for 3 weeks) corrected adenosine-induced negative chronotropic effect in STZ-induced diabetic animals, but selenium supplementation had a minimal effect on carbachol-induced inotropic and chronotropic responses in the left and right atria [70]. In the aorta of STZ-induced diabetic rats, sodium selenate treatment (0.3 mg/kg/d for 4 weeks) can improve isoproterenol-induced relaxation and contraction responses and preserve the morphology of smooth muscle cells. This may be related to the regulation on MMP-2 activity and protein loss in aorta, as well as the inhibition of tissue nitrite and protein thiol oxidation. Pathway study revealed that selenium supplementation might improve endothelin-1, PKC, and cAMP production in the aorta [71]. Aydemir-Koksoy et al. found that treatment of sodium selenite (0.3 mg/kg/d) could prevent depression in the left ventricular development pressure and the rates of changes in developed pressure in STZ-induced diabetic rats, and this effect was much greater than antioxidant treatment using vitamin E combined with omega-3 fish oil. The increase of myocardial oxidized protein sulfhydryl and nitrite concentration in the heart of diabetic rats was normalized by selenium supplementation [72]. Mechanism study revealed that myocardial MMP-2 and TIMP-4 were normalized, and selenite treatment increased expression of Tnl and *α*-actin in the heart of diabetic mice [73]. Liu et al. also revealed that high glucose-induced cardiomyocyte apoptosis could be attenuated by selenium supplementation through regulating TLR-4/MyD-99 signalling pathway and ROS formation [74]. Inhibition of NF-*κ*B-mediated proinflammatory cytokine transcription and suppression of leukotriene pathway by sodium selenite treatment also contributed to the protective effect of selenium against diabetic cardiac hypertrophy [75]. Ng et al. observed that a water-soluble selenium-containing sugar rendered antioxidant activity in the aortae and prevented hyperglycaemia-induced endothelial dysfunction through reducing superoxide levels, as well as improving basal NO availability and vasoconstrictor prostanoids [76]. Combination of selenium with low-dose insulin can restore PI3K-mediated GLUT4 in cardiac muscle, which reduced damage and dysfunction of myocardial cells in STZ-induced diabetic rats [45].

Kornhauser et al. observed that, in type 2 diabetic patients, plasma selenium level was reduced. Serum concentration of GPX was significantly lower in diabetic patients with microalbuminuria than in those without nephropathy. Notably, microalbuminuria was negatively correlated with plasma level of selenium and GPX in patients with type 2 diabetes [77]. The role of selenium in diabetic nephropathy was evident by the observation that animal fed with selenium-deficient diet developed albuminuria and glomerular sclerosis as well as increased expression of TGF-*β*1 mRNA. Supplementation of selenium (0.27 mg/kg Se in diet) in the form of sodium selenite in diabetic rats improved glomerular sclerosis and tubulointerstitium [78]. Roy et al. observed that sodium selenate treatment (16 *μ*mol/kg) could improve serum creatinine, urea, and albumin levels, as well as the renal antioxidant enzyme activities, such as superoxide dismutase (SOD), catalase, and GSH in STZ-induced diabetic rats. Selenate treatment could reduce lipid peroxidation and TGF-*β*1 in the diabetic rat kidney and improve cellular architecture of the kidney. This may lead to reduce apoptotic renal cells in diabetic mice [79]. In contrast, study from Bas et al. found that sodium selenite treatment (1 mg/kg for 28 days) had a minimal effect on diabetes-mediated toxicity in kidneys through improving lead nitrate-induced nephrotoxicity in nondiabetic animals [80].

Intraperitoneal injection of sodium selenite (5 *μ*mol/kg/day) for 4 weeks did not significantly improve high blood glucose and body weight loss in diabetic animals, but seemed to improve diabetes-induced structural alterations in the mandible [81]. Ozdemir et al. observed in STZ-induced diabetes that intraperitoneal injection of 5 *μ*mol/kg/d for 4 weeks could prevent deterioration of structural and ultrastructural changes in the long bones of diabetic rats [82].

In type 1 diabetic rats induced by STZ injection, treatment of sodium selenite (5 *μ*g/kg/d, intraperitoneal injection for 4 weeks) could significantly improve liver antioxidant enzymes in diabetic rats. The ultrastructure of the liver tissue, including variation in staining quality of hepatocyte nuclei, density, and eosinophilia of the cytoplasm, focal sinusoidal dilatation and congestion, and number of abnormal mitochondria, was normalized by sodium selenite treatment [83]. Intraperitoneal injection of sodium selenite (1.5 mg/kg/d for 4 weeks) could improve the liver function of STZ-induced diabetic animals and increase the hepatic expression of superoxide dismutase, reduce glutathione, lactate dehydrogenase, pyruvate kinase, and hexokinase, which rendered inhibition to NO, MDA, and phosphoenolpyruvate carboxykinase (PEPCK) in the liver [84]. Supplementation of selenium in the form of sodium selenite (1 ppm in drinking water) reduced aspartate aminotransferase (AST), alanine aminotransferase (ALT), and alkaline phosphatase (ALP) in diabetic rats, with a significant improvement in serum antioxidant enzymes and reduction of GSH level. Improvement of hepatic lipid accumulation and centrilobular hepatocyte degeneration was also observed [85]. In addition, treatment of sodium selenite (0.5 mg/kg/d for 4 weeks) could significantly reduce aldehyde oxidase and xanthine oxidase activities in the liver, but not in the kidney or heart, which might be associated with improvement of total antioxidant status after selenium supplementation [86].

## 4. The Role of Selenium in Treatment of Hyperphenylalaninemia

Phenylketonuria (PKU) is a born error in amino acid metabolism which leads to mildly or strongly elevated concentrations of the amino acid phenylalanine in the blood. PKU is the major cause of hyperphenylalaninemia. Studies have supported that in patients with PKU, the antioxidant defence in plasma and erythrocytes was decreased, which can be due to the secondary deprivation of micronutrients [87]. An observation from 156 patients with hyperphenylalaninemia showed that selenium was diminished in 25% of the subjects, 95% of which exhibited phenylketonuric phenotype [88]. The reason of low plasma selenium could be diet related, as PKU patients are often required to take natural protein and phenylalanine-restricted diet, which brings risk of low selenium intake [89]. Plasma level of selenium was significantly lower in patients with phenylketonuria or milder hyperphenylalaninemia, consistent with low total antioxidant status. The plasma selenium was correlated with erythrocyte GPX activity, which was lower in phenylketonuria, but inversely associated with free triiodothyronine and thyroxine [90, 91]. In contrast, Artuch and colleagues showed that plasma selenium concentration in patients with phenylketonuria had no different change compared with the healthy population [92]. In maternal Czech women with hyperphenylalaninemia, reduction of serum and urinary selenium level was observed [93]. Selenium deficiency led to defective GPX activities and consequently an increased level of MDA and organic hydroperoxides in the serum [94]. Further study showed that selenium deficiency in phenylketonuria might be the aetiology of dysrhythmia and cardiac dysfunction [95, 96]. Selenium deficiency in phenylketonuria might cause reduced response to OKT3 mitogenesis via T-cell antigen receptor complex (TCR/CD3) [97]. Gassio et al. reported a consistent observation of low-serum selenium level in patients with phenylketonuria and found that selenium concentration was associated with worsen Conners' Continuous Performance Test measures (more omission errors, fluctuating attention and inconsistency of response times, and slowing reaction time as the test progressed) [98]. However, another study showed that the neuropsychological disturbance in phenylketonuria patients might be independent to selenium level, as plasma selenium seems to be normal in patients, while patients with lower selenium GPX had more severe neuropsychological disturbances [99].

The decreased level of serum selenium in phenylketonuric patients did not improve by dietotherapy [100, 101]. A study in Czech patients with phenylketonuria and hyperphenylalaninemia showed that controlled diet with low protein may cause serum selenium deficiency in adults, while prealbumin, zinc, and iron remained unchanged [102]. Consistent observation was found in another 12-year study on selenium status in 78 phenylketonuric children (aged 1–16) [103]. In patients with phenylalanine-restricted diet, intake of sodium selenite (115 *μ*g/d) for 3 months could increase selenium level in plasma and blood cells and improve plasma GPX activity and left ventricular cardiac index, which led to decrease of thyroxin, free thyroxin, reverse triiodthyronin, TC, mean erythrocyte and thrombocyte volume, and lymphocytic CD2 expressions [104]. In patients with phenylalanine-restricted diet, selenium supplementation (1 *μ*g/kg/d for 3 weeks) could reduce both concentrations of prohormone thyroxine (T4) and metabolic inactive reverse triiodothyronine (rT3), which could be probably due to the increase in activity of type I 5′-deiodinase [105, 106].

A pilot observation on 5 patients was conducted by Lombeck and colleagues, who showed that supplementation of selenium (45 *μ*g/d) could render a normal selenium level in blood of phenylketonuric patients after a 4-week treatment, though GPX activity was only partially normalized [107]. However, Zachara et al. found that GPX activity in red blood cells of patients with phenylketonuria well indicated the functional restoration of selenium supplementation [108]. A possible mechanism underlying this discrepancy may be understood from the observation that selenium supplementation (0.13 mmol/kg/day) could only result in a short-term (within 10 days) but not long-term increase of plasma selenium level [109]. Using a special formula containing 31.5 *μ*g/d selenium and 98 mg/d L-carnitine reduced lipid peroxidation and protein oxidative damage and improved GPX activity in phenylketonuric patients, indicating that selenium supplementation was important for the amelioration of neurological symptoms of phenylketonuria via regulating oxidative stress pathways [110]. Alves observed in a clinical study of phenylketonuric children that selenium supplementation could significantly increase serum selenium and GPX in erythrocytes, which in turn reduced serum concentration of free thyroxin and improved patient conditions [111].

## 5. Discussion and Conclusion

### 5.1. Toxicity of Selenium

Although the reviewed studies and some other ongoing investigations have been providing mounting evidence on the beneficial role of selenium in both healthy people and patients, it is necessary to pay attention to its toxicity which is probably due to overdose of daily intake from food and water. As selenium can be accumulated through the food chain, selenium contamination, especially in the aquatic environment, can lead to enrichment of selenium speciation, such as Se(IV), Se(VI), and selenomethionine in plants and fishes [112]. These selenium species may cause direct toxicity [113], which may be related to the induction of ROS-associated oxidative stress [114]. What is more, aquatic organisms exposed under high-dose selenium are taking a risk of organ damage and genome mutation [115, 116], making them susceptible in safety as human food. In this case, environmental selenium accumulation may bring primary risk (for selenium enrichment) and secondary risk (from unknown mutation-borne food toxicity) to the human body, and this yielded attempt to set up criteria for allowable selenium level in aquatic system by different organizations (Table 1, adopted from review by Sharma et al. [117]).

Chronic exposure of environment selenium has been demonstrated to be a high risk factor of health in human population. Selenium overdose in humans may develop selenosis [118, 119], though quite rare, and is possible to cause amyotrophic lateral sclerosis [120] regardless of races and ethnicities [121–123]. Mechanistically, cellular exposure of high-dose selenium can cause elevation of intracellular ROS, which is considered as the main mediator of selenium-induced cell toxicity [124]. Though selenium is overall regarded as an essential factor of antioxidant enzyme production, chemically, it is capable to react and form intramolecular disulfide bond (S-Se) with essential thiol groups, or cysteine resides in the substrates [125] and indirectly generates ROS. The increasing oxidizing cellular environment may then cause DNA damage and genome instability, leading to initiation of cell apoptosis [126, 127]. The oxidative stress-involving selenium toxicity might therefore lead to impaired immune function, cytotoxicity, genotoxicity, and carcinogenesis [128–130]. Overdose of selenium can be lethal; as shown in rats, the LD_50_ values were 7, 138, and 6700 mg Se/kg.bw for selenite, selenium sulphide, and elemental Se, respectively [131]. In humans, daily intake of selenium at 4.9 mg/person/day was considered toxic in Chinese and Indian populations [4, 132, 133]. The issue of dose of intake shall be therefore taken into serious account when using selenium as a nutritional or therapeutic agent.

### 5.2. Dose Recommendation for Selenium Intake

Given the double-sword nature of selenium intake, the daily dose of intake is quite a critical issue for selenium as either nutritional supplements or therapeutics. In spite of the official guideline for the use of selenium as beneficial supplements in patients is not yet developed, a lot of efforts have been made to specify the selenium intake in a healthy population. The World Health Organization (WHO) has made recommendation on the dose of selenium for adults to be 30 to 40 *μ*g/day and stated that daily intake up to 400 *μ*g selenium shall be considered safe [134]. Recommended dose of selenium varies in different countries in consideration of differences in geographical and racial natures as well as in living styles of particular populations.  Table 2 summarized recommendations on daily dose of selenium from official and/or nonofficial organizations in various regions, which was adopted from a recent review by Kieliszek and Blazejak [134]. Optimal dose of selenium intake in patients with metabolic diseases is difficult to estimate, but from several studies we retrieved in this review, it seems that daily supplementation of 31.5–200 *μ*g Se is beneficial. There are not much literature for reference, since the limited amount of studies with inconsistent data quality, though most of these studies indicated patients with metabolic disorders, might need to take higher dose of selenium than the healthy population (82.4–200 *μ*g).

### 5.3. Proposed Mechanism of Action

**Figure 1:**
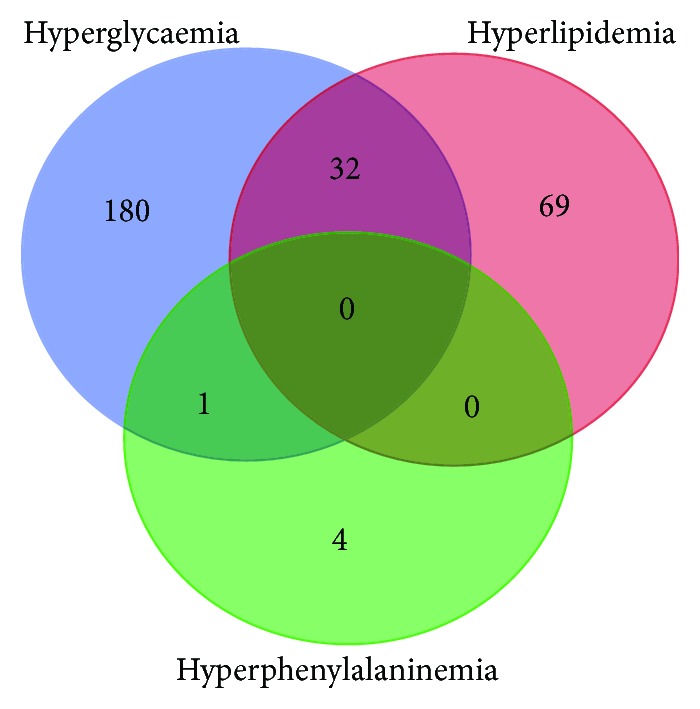
Venn diagram of interacting genes among various metabolic disorders. Disease-related genes were retrieved from the NCBI database, and Venn diagram was created to find any common gene involved in different metabolic diseases. 213 genes for hyperglycaemia (shown in blue circle), 101 genes for hyperlipidemia (shown in red circle), and 5 genes for hyperphenylalaninemia (shown in green circle) were collected. Hyperlipidemia and hyperglycemia share 32 common disease-related genes, while there is only one gene overlap between the groups of hyperglycaemia and hyperphenylalaninemia. No common gene is observed for the three diseases. Detail lists of genes were shown in Table S1.

Metabolic disorders are a series of diseases resulting from breakdown of internal homeostasis of the human body, which involves an infinite cycle of energy synthesis and waste production. Major metabolic dysregulation including hyperglycaemia, hyperlipidaemia, and hyperphenylalaninemia causes illness in multiple organs including the livers, kidney, and heart, leading to a series of diseases such as obesity, diabetes, phenylketonuria, and atherosclerosis. To further understand the molecular function of selenium, we retrieved genes related to multiple disorders (Table S1). Hyperphenylalaninemia seems to be caused by independent mechanism, while hyperglycaemia and hyperlipidaemia share a series of related genes (Figure 1, Table S2). By searching stitch 4.0 and STRING database, we found that selenium interacts with a series of selenoproteins, which secondarily interact with a series of proteins (Table S3). Particularly, it was noticed that xanthine dehydrogenase (XDH) is the interacting protein that connects the pathogenesis with molecular action of selenium. As superoxide-producing enzyme XDH and its converted form xanthine oxidase (XO) have been found increased in metabolic diseases [135, 136]. Although there has not been direct evidence showing that selenium treatment suppresses XDH and XO, a previous study showing the inverse correlation between selenium-associated GPX enzyme level with XDH level in diabetic rats [65] suggested that selenium may be related to the activity of xanthine metabolism. Future original study may focus on the role of XDH/XO system in the therapeutic role of selenium in metabolic disorders via GPX system.

### 5.4. Future Direction

Selenium has been proposed to be beneficial supplements for human health. Although high dose of selenium can definitely cause toxicity, the rational intake of selenium shall be safe and useful to not only healthy population but also patients with metabolic diseases. Efforts have been made to understand the action of selenium in metabolic diseases, and more clinical-relevant studies in the future are highly expected. The dose and form of selenium given to metabolic patients shall be standardised by official guidelines. Efficacy and safety of selenium supplementation in improving metabolic disorders shall be proven by long-term follow-up in patients. In addition, the mechanism underlying the action of selenium, which may be dependent or independent to internal antioxidant defence system, shall be studied by more experimental investigations.

In conclusion, we systemically reviewed the role of selenium as an antioxidant in various metabolic disorders. Selenium deficiency is observed in multiple metabolic diseases, including hyperglycaemia, hyperlipidaemia, and hyperphenylalaninemia. Supplementation of selenium may improve atherosclerosis, hypercholesterolemia, type 1 diabetes mellitus, and phenylketonuria, but its action remains controversial for type 2 diabetes mellitus. While regulation of hyperphenylalaninemia may go through an independent mechanism, hyperglycaemia and hyperlipidaemia may have shared mechanisms with a series of common genes involved. Toxicity of selenium was highlighted, and the window of selenium between beneficial and toxic doses shall be paid attention to recommend a proper dose of administration. The antioxidant role of selenium in metabolic diseases may be highlighted with the prediction that selenium-related proteins may interact with xanthine metabolism and superoxide-producing enzymes in metabolic diseases. Our study indicates the therapeutic potential of selenium supplementation as an antioxidant in the treatment of metabolic disorders.

## Figures and Tables

**Table 1:** Allowable Se level in aquatic systems^∗^

Organization	Criteria
United States Environmental Protection Agency	Se(IV) ≤ 257 *μ*g/L; Se(VI) ≤ 417 *μ*g/L
The French Institute of Industrial Environment and Risks	0.88 *μ*g/L as probable no effect concentration (PNEC) for direct chronic effect; 0.97 *μ*g/L as no observable effect concentration
British Columbia	PNEC = 2 *μ*g/L for freshwaters and marine environment
Environment Canada	PNEC = 1 *μ*g/L for freshwaters

^∗^Adopted from a review by Sharma et al. [117].

**Table 2:** Recommendations on daily dose of selenium^∗^

Counties/regions	Recommendations
Czech Republic	10–25 *μ*g/day
China	7–4990 *μ*g/day
Venezuela	200–350 *μ*g/day
Poland	30–40 *μ*g/day
Austria	48 *μ*g/day
Great Britain	34 *μ*g/day
USA	40–70 *μ*g/day for men; 45–55 *μ*g/day for women

^∗^Adopted from a recent review by Kieliszek and Blazejak [134].

## Supplementary Materials

Supplementary Materials Table S1: genes involved in major metabolic disorders. Table S2: genes of interests. Table S3: predicted partner genes of selenium proteins.

## References

[1] Rayman M. P. (2012). Selenium and human health. *Lancet*.

[2] Park K., Rimm E., Siscovick D., Spiegelman D., Morris J. S., Mozaffarian D. (2011). Demographic and lifestyle factors and selenium levels in men and women in the U.S. *Nutrition Research and Practice*.

[3] Wrobel J. K., Power R., Toborek M. (2016). Biological activity of selenium: revisited. *IUBMB Life*.

[4] Tinggi U. (2008). Selenium: its role as antioxidant in human health. *Environmental Health and Preventive Medicine*.

[5] Wei J., Zeng C., Gong Q. Y., Li X. X., Lei G. H., Yang T. B. (2015). Associations between dietary antioxidant intake and metabolic syndrome. *PLoS One*.

[6] Zulet M. A., Puchau B., Hermsdorff H. H., Navarro C., Martinez J. A. (2009). Dietary selenium intake is negatively associated with serum sialic acid and metabolic syndrome features in healthy young adults. *Nutrition Research*.

[7] Fulop P., Seres I., Jenei Z., Juhasz I., Paragh G. (2013). Increased hair selenium concentration in hyperlipidemic patients. *Journal of Cellular and Molecular Medicine*.

[8] Karita K., Yamanouchi Y., Takano T., Oku J., Kisaki T., Yano E. (2008). Associations of blood selenium and serum lipid levels in Japanese premenopausal and postmenopausal women. *Menopause*.

[9] Coudray C., Roussel A. M., Mainard F., Arnaud J., Favier A. (1997). Lipid peroxidation level and antioxidant micronutrient status in a pre-aging population; correlation with chronic disease prevalence in a French epidemiological study (Nantes, France). *Journal of the American College of Nutrition*.

[10] El-Demerdash F. M., Nasr H. M. (2014). Antioxidant effect of selenium on lipid peroxidation, hyperlipidemia and biochemical parameters in rats exposed to diazinon. *Journal of Trace Elements in Medicine and Biology*.

[11] Sreekala S., Indira M. (2008). Effect of exogenous selenium on nicotine induced hyperlipidemia in rats. *Indian Journal of Physiology and Pharmacology*.

[12] da Rocha J. T., Speranca A., Nogueira C. W., Zeni G. (2009). Hypolipidaemic activity of orally administered diphenyl diselenide in triton wr-1339-induced hyperlipidaemia in mice. *The Journal of Pharmacy and Pharmacology*.

[13] Sartori Oliveira C. E., Pinton S., da Rocha J. T., Gai B. M., Nogueira C. W. (2016). The hypolipidemic action of a diet supplemented with p,p'-methoxyl-diphenyl diselenide is not directly related to its antioxidant property. *Canadian Journal of Physiology and Pharmacology*.

[14] Ibrahim H. A., Zhu Y., Wu C. (2012). Selenium-enriched probiotics improves murine male fertility compromised by high fat diet. *Biological Trace Element Research*.

[15] Kalkan Ucar S., Coker M., Sozmen E., Goksen Simsek D., Darcan S. (2010). An association among iron, copper, zinc, and selenium, and antioxidative status in dyslipidemic pediatric patients with glycogen storage disease types IA and III. *Journal of Trace Elements in Medicine and Biology*.

[16] Chan Y. H., Siu C. W., Yiu K. H. (2012). Adverse systemic arterial function in patients with selenium deficiency. *The Journal of Nutrition, Health & Aging*.

[17] Al-Rasheed N. M., Attia H. A., Mohamed R. A., Al-Rasheed N. M., Al-Amin M. A. (2013). Preventive effects of selenium yeast, chromium picolinate, zinc sulfate and their combination on oxidative stress, inflammation, impaired angiogenesis and atherogenesis in myocardial infarction in rats. *Journal of Pharmacy & Pharmaceutical Sciences*.

[18] Stranges S., Laclaustra M., Ji C. (2010). Higher selenium status is associated with adverse blood lipid profile in British adults. *The Journal of Nutrition*.

[19] Kuklinski B., Zimmermann R., Ruhlmann C., Herzfeld A. (1990). Latent antioxidant deficiency in the east german population--causes and clinical significance. II. *Zeitschrift fur die gesamte innere Medizin und ihre Grenzgebiete*.

[20] Delattre J., Lepage S., Jaudon M. C., Bruckert E., Assogba U., Bonnefont-Rousselot D. (1998). The plasma antioxidant status and trace elements in patients with familial hypercholesterolemia treated with LDL-apheresis. *Annales Pharmaceutiques Francaises*.

[21] Lepage S., Bonnefont-Rousselot D., Bruckert E. (1996). Antioxidant status of hypercholesterolemic patients treated with LDL apheresis. *Cardiovascular Drugs and Therapy*.

[22] Linseisen J., Wilhelm M., Hoffmann J., Hailer S., Keller C., Wolfram G. (1999). Acute effects of LDL-apheresis on cholesterol oxidation products and antioxidants in plasma and lipoproteins of patients with familial hypercholesterolemia. *European Journal of Medical Research*.

[23] Galicka-Latala D., Jarzabek Z. (1992). Selenium and malondialdehyde levels in the blood serum of men with normo- and hypercholesterolemia. *Przeglad lekarski*.

[24] Dhingra S., Bansal M. P. (2006). Attenuation of LDL receptor gene expression by selenium deficiency during hypercholesterolemia. *Molecular and Cellular Biochemistry*.

[25] Dhingra S., Bansal M. P. (2005). Hypercholesterolemia and apolipoprotein b expression: regulation by selenium status. *Lipids in Health and Disease*.

[26] Dhingra S., Bansal M. P. (2006). Modulation of hypercholesterolemia-induced alterations in apolipoprotein B and HMG-CoA reductase expression by selenium supplementation. *Chemico-Biological Interactions*.

[27] Iizuka Y., Sakurai E., Tanaka Y. (2001). Effect of selenium on serum, hepatic and lipoprotein lipids concentration in rats fed on a high-cholesterol diet. *Yakugaku Zasshi*.

[28] Kaur H. D., Bansal M. P. (2009). Studies on HDL associated enzymes under experimental hypercholesterolemia: possible modulation on selenium supplementation. *Lipids in Health and Disease*.

[29] Kang B. P., Bansal M. P., Mehta U. (2000). Hyperlipidemia and type I 5′-monodeiodinase activity: regulation by selenium supplementation in rabbits. *Biological Trace Element Research*.

[30] Dhingra S., Singh U., Bansal M. P. (2003). Protective role of selenium status on T3/T4 kinetics in rats under hyperlipidemia. *Indian Journal of Biochemistry & Biophysics*.

[31] Gonca S., Ceylan S., Yardimoglu M. (2000). Protective effects of vitamin E and selenium on the renal morphology in rats fed high-cholesterol diets. *Pathobiology*.

[32] Cser A., Sziklai-Laszlo I., Menzel H., Lombeck I. (1993). Selenium status and lipoproteins in healthy and diabetic children. *Journal of Trace Elements and Electrolytes in Health and Disease*.

[33] Osterode W., Holler C., Ulberth F. (1996). Nutritional antioxidants, red cell membrane fluidity and blood viscosity in type 1 (insulin dependent) diabetes mellitus. *Diabetic Medicine*.

[34] Gumuslu S., Yargicoglu P., Agar A., Edremitlioglu M., Aliciguzel Y. (1997). Effect of cadmium on antioxidant status in alloxane-induced diabetic rats. *Biological Trace Element Research*.

[35] Sheng X. Q., Huang K. X., Xu H. B. (2005). Influence of alloxan-induced diabetes and selenite treatment on blood glucose and glutathione levels in mice. *Journal of Trace Elements in Medicine and Biology*.

[36] Guney M. (2012). Selenium-vitamin E combination modulates endometrial lipid peroxidation and antioxidant enzymes in streptozotocin-induced diabetic rat. *Biological Trace Element Research*.

[37] Sokmen B. B., Basaraner H., Yanardag R. (2012). Combined effects of treatment with vitamin C, vitamin E, and selenium on the skin of diabetic rats. *Human & Experimental Toxicology*.

[38] Satyanarayana S., Sekhar J. R., Kumar K. E., Shannika L. B., Rajanna B., Rajanna S. (2006). Influence of selenium (antioxidant) on gliclazide induced hypoglycaemia/anti hyperglycaemia in normal/alloxan-induced diabetic rats. *Molecular and Cellular Biochemistry*.

[39] Atalay M., Bilginoglu A., Kokkola T., Oksala N., Turan B. (2011). Treatments with sodium selenate or doxycycline offset diabetes-induced perturbations of thioredoxin-1 levels and antioxidant capacity. *Molecular and Cellular Biochemistry*.

[40] Bajpai S., Mishra M., Kumar H. (2011). Effect of selenium on connexin expression, angiogenesis, and antioxidant status in diabetic wound healing. *Biological Trace Element Research*.

[41] Chen H., Qiu Q., Zou C., Dou L., Liang J. (2015). Regulation of hepatic carbohydrate metabolism by selenium during diabetes. *Chemico-Biological Interactions*.

[42] Barakat G. M., Moustafa M. E., Bikhazi A. B. (2012). Effects of selenium and exendin-4 on glucagon-like peptide-1 receptor, IRS-1, and Raf-1 in the liver of diabetic rats. *Biochemical Genetics*.

[43] Kahya M. C., Naziroglu M., Cig B. (2015). Melatonin and selenium reduce plasma cytokine and brain oxidative stress levels in diabetic rats. *Brain Injury*.

[44] Erbayraktar Z., Yilmaz O., Artmann A. T., Cehreli R., Coker C. (2007). Effects of selenium supplementation on antioxidant defense and glucose homeostasis in experimental diabetes mellitus. *Biological Trace Element Research*.

[45] Xu T. J., Yuan B. X., Zou Y. M. (2011). Effect of combination of insulin and selenium on insulin signal transduction in cardiac muscle of STZ-induced diabetic rats. *Yao xue xue bao*.

[46] Liu Y., Li C., Luo X. (2015). Characterization of selenium-enriched mycelia of *Catathelasma ventricosum* and their antihyperglycemic and antioxidant properties. *Journal of Agricultural and Food Chemistry*.

[47] Naziroglu M., Cay M. (2001). Protective role of intraperitoneally administered vitamin E and selenium on the antioxidative defense mechanisms in rats with diabetes induced by streptozotocin. *Biological Trace Element Research*.

[48] Anderson R. A., Roussel A. M., Zouari N., Mahjoub S., Matheau J. M., Kerkeni A. (2001). Potential antioxidant effects of zinc and chromium supplementation in people with type 2 diabetes mellitus. *Journal of the American College of Nutrition*.

[49] Stranges S., Marshall J. R., Natarajan R. (2007). Effects of long-term selenium supplementation on the incidence of type 2 diabetes: a randomized trial. *Annals of Internal Medicine*.

[50] Gonzalez de Vega R., Fernandez-Sanchez M. L., Fernandez J. C., Alvarez Menendez F. V., Sanz-Medel A. (2016). Selenium levels and glutathione peroxidase activity in the plasma of patients with type II diabetes mellitus. *Journal of Trace Elements in Medicine and Biology*.

[51] Mueller A. S., Bosse A. C., Most E., Klomann S. D., Schneider S., Pallauf J. (2009). Regulation of the insulin antagonistic protein tyrosine phosphatase 1B by dietary Se studied in growing rats. *The Journal of Nutritional Biochemistry*.

[52] Wang X. D., Vatamaniuk M. Z., Wang S. K., Roneker C. A., Simmons R. A., Lei X. G. (2008). Molecular mechanisms for hyperinsulinaemia induced by overproduction of selenium-dependent glutathione peroxidase-1 in mice. *Diabetologia*.

[53] Zhou J., Xu G., Bai Z. (2015). Selenite exacerbates hepatic insulin resistance in mouse model of type 2 diabetes through oxidative stress-mediated JNK pathway. *Toxicology and Applied Pharmacology*.

[54] Faghihi T., Radfar M., Barmal M. (2014). A randomized, placebo-controlled trial of selenium supplementation in patients with type 2 diabetes: effects on glucose homeostasis, oxidative stress, and lipid profile. *American Journal of Therapeutics*.

[55] Ozturk Z., Gurpinar T., Vural K., Boyacioglu S., Korkmaz M., Var A. (2015). Effects of selenium on endothelial dysfunction and metabolic profile in low dose streptozotocin induced diabetic rats fed a high fat diet. *Biotechnic & Histochemistry*.

[56] Ren D., Hu Y., Luo Y., Yang X. (2015). Selenium-containing polysaccharides from Ziyang green tea ameliorate high-fructose diet induced insulin resistance and hepatic oxidative stress in mice. *Food & Function*.

[57] Tanko Y., Jimoh A., Ahmed A. (2017). Effects of selenium yeast on blood glucose and antioxidant biomarkers in cholesterol fed diet induced type 2 diabetes mellitus in wistar rats. *Nigerian Journal of Physiological Sciences*.

[58] Al-Saleh E., Nandakumaran M., Al-Shammari M., Al-Harouny A. (2004). Maternal-fetal status of copper, iron, molybdenum, selenium and zinc in patients with gestational diabetes. *The Journal of Maternal-Fetal & Neonatal Medicine*.

[59] Hawkes W. C., Alkan Z., Lang K., King J. C. (2004). Plasma selenium decrease during pregnancy is associated with glucose intolerance. *Biological Trace Element Research*.

[60] Bo S., Lezo A., Menato G. (2005). Gestational hyperglycemia, zinc, selenium, and antioxidant vitamins. *Nutrition*.

[61] Uusitalo L., Kenward M. G., Virtanen S. M. (2008). Intake of antioxidant vitamins and trace elements during pregnancy and risk of advanced β cell autoimmunity in the child. *The American Journal of Clinical Nutrition*.

[62] Guney M., Erdemoglu E., Mungan T. (2011). Selenium-vitamin e combination and melatonin modulates diabetes-induced blood oxidative damage and fetal outcomes in pregnant rats. *Biological Trace Element Research*.

[63] Asemi Z., Jamilian M., Mesdaghinia E., Esmaillzadeh A. (2015). Effects of selenium supplementation on glucose homeostasis, inflammation, and oxidative stress in gestational diabetes: randomized, double-blind, placebo-controlled trial. *Nutrition*.

[64] Naziroglu M. (2003). Enhanced testicular antioxidant capacity in streptozotocin-induced diabetic rats: protective role of vitamins C and E and selenium. *Biological Trace Element Research*.

[65] Aliciguzel Y., Ozen I., Aslan M., Karayalcin U. (2003). Activities of xanthine oxidoreductase and antioxidant enzymes in different tissues of diabetic rats. *The Journal of Laboratory and Clinical Medicine*.

[66] Liu Y., Sun J., Rao S. (2013). Antidiabetic activity of mycelia selenium-polysaccharide from *Catathelasma ventricosum* in STZ-induced diabetic mice. *Food and Chemical Toxicology*.

[67] Dkhil M. A., Zrieq R., Al-Quraishy S., Abdel Moneim A. E. (2016). Selenium nanoparticles attenuate oxidative stress and testicular damage in streptozotocin-induced diabetic rats. *Molecules*.

[68] Faure P. (2003). Protective effects of antioxidant micronutrients (vitamin E, zinc and selenium) in type 2 diabetes mellitus. *Clinical Chemistry and Laboratory Medicine*.

[69] Ayaz M., Can B., Ozdemir S., Turan B. (2002). Protective effect of selenium treatment on diabetes-induced myocardial structural alterations. *Biological Trace Element Research*.

[70] Gur S. (2004). Effects of sodium selenate treatment on altered responses of left and right atria from streptozotocin-induced diabetic rats. *Journal of Cardiovascular Pharmacology*.

[71] Zeydanli E. N., Bilginoglu A., Tanriverdi E., Gurdal H., Turan B. (2010). Selenium restores defective beta-adrenergic receptor response of thoracic aorta in diabetic rats. *Molecular and Cellular Biochemistry*.

[72] Aydemir-Koksoy A., Turan B. (2008). Selenium inhibits proliferation signaling and restores sodium/potassium pump function of diabetic rat aorta. *Biological Trace Element Research*.

[73] Aydemir-Koksoy A., Bilginoglu A., Sariahmetoglu M., Schulz R., Turan B. (2010). Antioxidant treatment protects diabetic rats from cardiac dysfunction by preserving contractile protein targets of oxidative stress. *The Journal of Nutritional Biochemistry*.

[74] Liu Z. W., Zhu H. T., Chen K. L., Qiu C., Tang K. F., Niu X. L. (2013). Selenium attenuates high glucose-induced ros/tlr-4 involved apoptosis of rat cardiomyocyte. *Biological Trace Element Research*.

[75] Dhanya B. L., Swathy R. P., Indira M. (2014). Selenium downregulates oxidative stress-induced activation of leukotriene pathway in experimental rats with diabetic cardiac hypertrophy. *Biological Trace Element Research*.

[76] Ng H. H., Leo C. H., O'Sullivan K. (2017). 1,4-anhydro-4-seleno-d-talitol (setal) protects endothelial function in the mouse aorta by scavenging superoxide radicals under conditions of acute oxidative stress. *Biochemical Pharmacology*.

[77] Kornhauser C., Garcia-Ramirez J. R., Wrobel K., Perez-Luque E. L., Garay-Sevilla M. E., Wrobel K. (2008). Serum selenium and glutathione peroxidase concentrations in type 2 diabetes mellitus patients. *Primary Care Diabetes*.

[78] Reddi A. S., Bollineni J. S. (2001). Selenium-deficient diet induces renal oxidative stress and injury via TGF-β1 in normal and diabetic rats. *Kidney International*.

[79] Roy S., Dontamalla S. K., Mondru A. K., Sannigrahi S., Veerareddy P. R. (2011). Downregulation of apoptosis and modulation of TGF-β1 by sodium selenate prevents streptozotocin-induced diabetic rat renal impairment. *Biological Trace Element Research*.

[80] Bas H., Kalender Y. (2016). Nephrotoxic effects of lead nitrate exposure in diabetic and nondiabetic rats: involvement of oxidative stress and the protective role of sodium selenite. *Environmental Toxicology*.

[81] Delilbasi C., Demiralp S., Turan B. (2002). Effects of selenium on the structure of the mandible in experimental diabetics. *Journal of Oral Science*.

[82] Ozdemir S., Ayaz M., Can B., Turan B. (2005). Effect of selenite treatment on ultrastructural changes in experimental diabetic rat bones. *Biological Trace Element Research*.

[83] Can B., Ulusu N. N., Kilinc K., Leyla Acan N., Saran Y., Turan B. (2005). Selenium treatment protects diabetes-induced biochemical and ultrastructural alterations in liver tissue. *Biological Trace Element Research*.

[84] Aly H. F., Mantawy M. M. (2012). Comparative effects of zinc, selenium and vitamin E or their combination on carbohydrate metabolizing enzymes and oxidative stress in streptozotocin induced-diabetic rats. *European Review for Medical and Pharmacological Sciences*.

[85] Zou C., Qiu Q., Chen H., Dou L., Liang J. (2015). Hepatoprotective effects of selenium during diabetes in rats. *Human & Experimental Toxicology*.

[86] Ghaffari T., Nouri M., Saei A. A., Rashidi M. R. (2012). Aldehyde and xanthine oxidase activities in tissues of streptozotocin-induced diabetic rats: effects of vitamin E and selenium supplementation. *Biological Trace Element Research*.

[87] Ribas G. S., Sitta A., Wajner M., Vargas C. R. (2011). Oxidative stress in phenylketonuria: what is the evidence?. *Cellular and Molecular Neurobiology*.

[88] Crujeiras V., Aldamiz-Echevarria L., Dalmau J. (2015). Vitamin and mineral status in patients with hyperphenylalaninemia. *Molecular Genetics and Metabolism*.

[89] Rocha J. C., Martins M. J. (2012). Oxidative stress in phenylketonuria: future directions. *Journal of Inherited Metabolic Disease*.

[90] van Bakel M. M., Printzen G., Wermuth B., Wiesmann U. N. (2000). Antioxidant and thyroid hormone status in selenium-deficient phenylketonuric and hyperphenylalaninemic patients. *The American Journal of Clinical Nutrition*.

[91] Jochum F., Terwolbeck K., Meinhold H., Behne D., Menzel H., Lombeck I. (1997). Effects of a low selenium state in patients with phenylketonuria. *Acta Paediatrica*.

[92] Artuch R., Colome C., Sierra C. (2004). A longitudinal study of antioxidant status in phenylketonuric patients. *Clinical Biochemistry*.

[93] Hyanek J., Bendl J., Zeman J. (1996). Maternal hyperphenylalaninemia in a population of healty Czech women. 18 years’ experience with mass screening, diet therapy and metabolic monitoring. *Casopis Lekaru Ceskych*.

[94] Wilke B. C., Vidailhet M., Richard M. J., Ducros V., Arnaud J., Favier A. (1993). Trace elements balance in treated phenylketonuria children. Consequences of selenium deficiency on lipid peroxidation. *Archivos latinoamericanos de nutricion*.

[95] Greeves L. G., Carson D. J., Craig B. G., McMaster D. (1990). Potentially life-threatening cardiac dysrhythmia in a child with selenium deficiency and phenylketonuria. *Acta Paediatrica Scandinavica*.

[96] Gordon S. J., Latham S. C., Spink J. D., Galbraith A. J. (1991). Assessment of cardiac function by M-mode echocardiography in selenium-deficient phenylketonuric children. *Journal of Paediatrics and Child Health*.

[97] Collins R. J., Boyle P. J., Clague A. E., Barr A. E., Latham S. C. (1991). In vitro okt3-induced mitogenesis in selenium-deficient patients on a diet for phenylketonuria. *Biological Trace Element Research*.

[98] Gassio R., Artuch R., Vilaseca M. A., Fuste E., Colome R., Campistol J. (2008). Cognitive functions and the antioxidant system in phenylketonuric patients. *Neuropsychology*.

[99] Sierra C., Vilaseca M. A., Moyano D. (1998). Antioxidant status in hyperphenylalaninemia. *Clinica Chimica Acta*.

[100] Lombeck I., Kasperek K., Feinendegen L. E., Bremer H. J. (1975). Serum-selenium concentrations in patients with maple-syrup-urine disease and phenylketonuria under dieto-therapy. *Clinica Chimica Acta*.

[101] McKenzie R. L., Rea H. M., Thomson C. D., Robinson M. F. (1978). Selenium concentration and glutathione peroxidase activity in blood of New Zealand infants and children. *The American Journal of Clinical Nutrition*.

[102] Prochazkova D., Jarkovsky J., Vinohradska H., Konecna P., Machacova L., Dolezel Z. (2013). Controlled diet in phenylketonuria and hyperphenylalaninemia may cause serum selenium deficiency in adult patients: the Czech experience. *Biological Trace Element Research*.

[103] Evans S., Daly A., MacDonald J. (2014). The micronutrient status of patients with phenylketonuria on dietary treatment: an ongoing challenge. *Annals of Nutrition & Metabolism*.

[104] Kauf E., Seidel J., Winnefeld K. (1997). Selenium in phenylketonuria patients. Effects of sodium selenite administration. *Medizinische Klinik*.

[105] Calomme M., Vanderpas J., Francois B. (1995). Effects of selenium supplementation on thyroid hormone metabolism in phenylketonuria subjects on a phenylalanine restricted diet. *Biological Trace Element Research*.

[106] Calomme M. R., Vanderpas J. B., Francois B. (1995). Thyroid function parameters during a selenium repletion/depletion study in phenylketonuric subjects. *Experientia*.

[107] Lombeck I., Kasperek K., Bachmann D., Feinendegen L. E., Bremer H. J. (1980). Selenium requirements in patients with inborn errors of amino acid metabolism and selenium deficiency. *European Journal of Pediatrics*.

[108] Zachara B. A., Wasowicz W., Gromadzinska J., Sklodowska M., Cabalska B. (1987). Red blood cell glutathione peroxidase activity as a function of selenium supplementation in dietary treated children with phenylketonuria. *Biomedica Biochimica Acta*.

[109] Wilke B. C., Vidailhet M., Favier A. (1992). Selenium, glutathione peroxidase (gsh-px) and lipid peroxidation products before and after selenium supplementation. *Clinica Chimica Acta*.

[110] Sitta A., Vanzin C. S., Biancini G. B. (2011). Evidence that L-carnitine and selenium supplementation reduces oxidative stress in phenylketonuric patients. *Cellular and Molecular Neurobiology*.

[111] Alves M. R., Starling A. L., Kanufre V. C. (2012). Selenium intake and nutritional status of children with phenylketonuria in Minas Gerais, Brazil. *Jornal de Pediatria*.

[112] Li W. H., Ju Y. R., Liao C. M., Liao V. H. (2014). Assessment of selenium toxicity on the life cycle of *Caenorhabditis elegans*. *Ecotoxicology*.

[113] Hladun K. R., Smith B. H., Mustard J. A., Morton R. R., Trumble J. T. (2012). Selenium toxicity to honey bee (*Apis mellifera* L.) pollinators: effects on behaviors and survival. *PLoS One*.

[114] Franz E. D., Wiramanaden C. I., Janz D. M., Pickering I. J., Liber K. (2011). Selenium bioaccumulation and speciation in *Chironomus dilutus* exposed to water-borne selenate, selenite, or seleno-DL-methionine. *Environmental Toxicology and Chemistry*.

[115] Chapman P. M. (2009). Is selenium a global contaminant of potential concern?. *Integrated Environmental Assessment and Management*.

[116] Misra S., Hamilton C., Niyogi S. (2012). Induction of oxidative stress by selenomethionine in isolated hepatocytes of rainbow trout (*Oncorhynchus mykiss*). *Toxicology In Vitro*.

[117] Sharma V. K., McDonald T. J., Sohn M., Anquandah G. A. K., Pettine M., Zboril R. (2017). Assessment of toxicity of selenium and cadmium selenium quantum dots: a review. *Chemosphere*.

[118] Lenz M., Lens P. N. (2009). The essential toxin: the changing perception of selenium in environmental sciences. *The Science of the Total Environment*.

[119] Lee K. H., Jeong D. (2012). Bimodal actions of selenium essential for antioxidant and toxic pro-oxidant activities: the selenium paradox (review). *Molecular Medicine Reports*.

[120] Estevez A. O., Morgan K. L., Szewczyk N. J., Gems D., Estevez M. (2014). The neurodegenerative effects of selenium are inhibited by foxo and PINK1/PTEN regulation of insulin/insulin-like growth factor signaling in *Caenorhabditis elegans*. *Neurotoxicology*.

[121] Roman M., Jitaru P., Barbante C. (2014). Selenium biochemistry and its role for human health. *Metallomics : integrated biometal science*.

[122] Vinceti M., Solovyev N., Mandrioli J. (2013). Cerebrospinal fluid of newly diagnosed amyotrophic lateral sclerosis patients exhibits abnormal levels of selenium species including elevated selenite. *Neurotoxicology*.

[123] Schomburg L. (2016). Dietary selenium and human health. *Nutrients*.

[124] Sun J., Zheng X., Li H. (2017). Monodisperse selenium-substituted hydroxyapatite: controllable synthesis and biocompatibility. *Materials science & engineering. C, Materials for biological applications*.

[125] Ganther H. E. (1999). Selenium metabolism, selenoproteins and mechanisms of cancer prevention: complexities with thioredoxin reductase. *Carcinogenesis*.

[126] Chung Y. W., Kim T. S., Lee S. Y. (2006). Selenite-induced apoptosis of osteoclasts mediated by the mitochondrial pathway. *Toxicology Letters*.

[127] Zhu Z., Jiang W., Ganther H. E., Ip C., Thompson H. J. (2000). *In vitro* effects of Se-allylselenocysteine and Se-propylselenocysteine on cell growth, DNA integrity, and apoptosis. *Biochemical Pharmacology*.

[128] Mostofa M. G., Hossain M. A., Siddiqui M. N., Fujita M., Tran L. S. (2017). Phenotypical, physiological and biochemical analyses provide insight into selenium-induced phytotoxicity in rice plants. *Chemosphere*.

[129] Prasad K. S., Selvaraj K. (2014). Biogenic synthesis of selenium nanoparticles and their effect on As(III)-induced toxicity on human lymphocytes. *Biological Trace Element Research*.

[130] Selvaraj V., Yeager-Armstead M., Murray E. (2012). Protective and antioxidant role of selenium on arsenic trioxide-induced oxidative stress and genotoxicity in the fish hepatoma cell line PLHC-1. *Environmental Toxicology and Chemistry*.

[131] Cummins L. M., Kimura E. T. (1971). Safety evaluation of selenium sulfide antidandruff shampoos. *Toxicology and Applied Pharmacology*.

[132] Combs G. F. (2001). Selenium in global food systems. *The British Journal of Nutrition*.

[133] Winkel L. H., Johnson C. A., Lenz M. (2011). Environmental selenium research: from microscopic processes to global understanding. *Environmental Science & Technology*.

[134] Kieliszek M., Blazejak S. (2016). Current knowledge on the importance of selenium in food for living organisms: a review. *Molecules*.

[135] Aydemir M., Ozturk N., Dogan S., Aslan M., Olgar Y., Ozdemir S. (2012). Sodium tungstate administration ameliorated diabetes-induced electrical and contractile remodeling of rat heart without normalization of hyperglycemia. *Biological Trace Element Research*.

[136] Schroder K., Vecchione C., Jung O. (2006). Xanthine oxidase inhibitor tungsten prevents the development of atherosclerosis in ApoE knockout mice fed a Western-type diet. *Free Radical Biology & Medicine*.

