# Supplementary material for: Supplementation of Micronutrient Selenium in Metabolic Diseases: Its Role as an Antioxidant
Source: Oxid Med Cell Longev. 2017 Dec 26;2017:7478523. doi: 10.1155/2017/7478523 (PMC5758946; doi:10.1155/2017/7478523)
Supplement: Supplementary Materials — Table S1: genes involved in major metabolic disorders. Table S2: genes of interests. Table S3: predicted partner genes of selenium proteins. [file 7478523.f1.docx]

**Table S1 Genes involved in major metabolic disorders**

| Metabolic disorder | Gene Involved |
| --- | --- |
| Hyperglycemia | ACE PPARD IL7R ECE1 ANXA2 KCNMA1 ROCK1 CXCL10 PROX1 EDN1 MUC22 PPARGC1A LOC102724584 ARRB2 SNAI1 HYMAI CACNA1D CYBA MAPK14 AOC3 KDR PYY3 NOX1 ANXA1 HMOX1 G6PC2 NTSR1 GLO1 ENTPD1 HNF1A NOS2 TRPM7 INS SIRT2 MEN1 DEAF1 WWTR1 FCGRT IL1A MTNR1B BRD2 SGK1 INVS CD59 TCF7L2 KCNMB1 GCG SETD7 DDOST PRDX6 SHC1 LZTS1 KRT8 CNDP1 CYC1 S1PR2 TGM2 PRKCB DEFB1 SMO CRYM LEPQTL1 MAPK1 CAPN10 IGF2 LRP1B ABCG8 ZNF295-AS1 MUC21 AHSG PTGS2 UCHL1 BIRC5 FABP1 NCF1 MLXIPL AKT2 H19 ABCC1 BOLA3 SPHKAP DPP4 IL1B IL18 PLTP S1PR1 SFR1 CYP2E1 CTH ADPGK PDX1 GRB2 IGFBP1 NPVF AGTR1 TXN GCH1 BTRC MYCBP2 CPT1A IL4 F3 TGFBRAP1 UCP3 FOXO3 IFI30 TLR2 PCK2 GCK GPX1 CELA1 TGFB1 KCNJ11 PTCH1 PMM2 SCN2B ABCC8 GHRL ICAM1 ADRA1D ERN1 BAMBI TNFRSF1B PIK3R2 MAPK3 UNC13B MCM8 ZFP57 AGT KRAS SMG1 GIP HSPG2 GCKR TRPC6 IL12A GLDC GPT RELA APOM PRKCA PRKCD CALCA CD40LG SI EIF2AK3 LHFP APOC1 NPHS1 RUNX1 PRKAA1 CTGF IL7 FGF14 CXCL8 APP RAN SLC2A1 GLRX5 GLP1R SAA1 MIR15B ERP44 XBP1 MIR182 MIR16-1 NSA2 TRPV1 MIR33B UCP2 PLAGL1 HMGB1 TFPI HBA1 GFAP |
| Hyperlipidemia | OSBPL10 SLC37A4 NPPA SERPINE1 RXRG MIR146A SLC25A13 APOA5 MMP3 HYPLIP2 LDLR FABP4 CYP2D6 SCD CYP2C19 ACSL3 ADRB3 CCR5 CD55 APOA2 OSM MMP14 LPA MIR370 MTRR NPPB CYP2C9 CPE MIR24-1 LCAT APOE C3 ARNT PTGS1 HAMP AGL NR1I2 DEFA1 FADS3 APOA1 CD36 ACSL5 CXCL12 CDKN2B LIPG C5AR2 HCRT FABP2 APOB PCSK9 CP CDH5 CCL2 NNMT SLC2A4 CYP3A4 PTH CYP7A1 CETP MTTP MIR122 VEGFB AS3MT PNPLA2 FN1 AHR USF1 NR1I3 |
| Hyperphenylalaninemia | PCBD1 DNAJC12 PTS PAH |

**Table S2 Genes of interests**

| Gene | Name |
| --- | --- |
| MYD88 | myeloid differentiation primary response 88(MYD88) |
| ENPP1 | ectonucleotide pyrophosphatase/phosphodiesterase 1(ENPP1) |
| VEGFA | vascular endothelial growth factor A(VEGFA) |
| HIF1A | hypoxia inducible factor 1 alpha subunit(HIF1A) |
| TXNIP | thioredoxin interacting protein(TXNIP) |
| APOC3 | apolipoprotein C3(APOC3) |
| REN | renin(REN) |
| SREBF1 | sterol regulatory element binding transcription factor 1(SREBF1) |
| LPL | lipoprotein lipase(LPL) |
| MTHFR | methylenetetrahydrofolate reductase(MTHFR) |
| IGF1 | insulin like growth factor 1(IGF1) |
| ADIPOQ | adiponectin, C1Q and collagen domain containing(ADIPOQ) |
| LEP | leptin(LEP) |
| TNF | tumor necrosis factor(TNF) |
| PPARG | peroxisome proliferator activated receptor gamma(PPARG) |
| SELE | selectin E(SELE) |
| NAMPT | nicotinamide phosphoribosyltransferase(NAMPT) |
| NOS3 | nitric oxide synthase 3(NOS3) |
| CRP | C-reactive protein(CRP) |
| XDH | xanthine dehydrogenase(XDH) |
| PPARA | peroxisome proliferator activated receptor alpha(PPARA) |
| G6PC | glucose-6-phosphatase catalytic subunit(G6PC) |
| AKR1B1 | aldo-keto reductase family 1 member B(AKR1B1) |
| IRS1 | insulin receptor substrate 1(IRS1) |
| FTO | FTO, alpha-ketoglutarate dependent dioxygenase(FTO) |
| CCL5 | C-C motif chemokine ligand 5(CCL5) |
| IL6 | interleukin 6(IL6) |
| RETN | resistin(RETN) |
| TLR4 | toll like receptor 4(TLR4) |
| NFKB1 | nuclear factor kappa B subunit 1(NFKB1) |
| PON1 | paraoxonase 1(PON1) |

**Table S3 Predicted partner genes of selenium-proteins**

| Gene | Name |
| --- | --- |
| VCP | valosin containing protein(VCP) |
| PINX1 | PIN2/TERF1 interacting, telomerase inhibitor 1(PINX1) |
| MYC | v-myc avian myelocytomatosis viral oncogene homolog(MYC) |
| PAK6 | p21 (RAC1) activated kinase 6(PAK6) |
| DERL1 | derlin 1(DERL1) |
| TGFB1I1 | transforming growth factor beta 1 induced transcript 1(TGFB1I1) |
| PIAS2 | protein inhibitor of activated STAT 2(PIAS2) |
| HAO2 | hydroxyacid oxidase 2(HAO2) |
| ABL1 | ABL proto-oncogene 1, non-receptor tyrosine kinase(ABL1) |
| SMAD3 | SMAD family member 3(SMAD3) |
| SMAD4 | SMAD family member 4(SMAD4) |
| KLK2 | kallikrein related peptidase 2(KLK2) |
| PIF1 | PIF1 5'-to-3' DNA helicase(PIF1) |
| ZMIZ1 | zinc finger MIZ-type containing 1(ZMIZ1) |
| MTOR | mechanistic target of rapamycin(MTOR) |
| MED1 | mediator complex subunit 1(MED1) |
| PTGES3 | prostaglandin E synthase 3(PTGES3) |
| GSTT1 | glutathione S-transferase theta 1(GSTT1) |
| PTEN | phosphatase and tensin homolog(PTEN) |
| HSP90AA1 | heat shock protein 90 alpha family class A member 1(HSP90AA1) |
| CREBBP | CREB binding protein(CREBBP) |
| GTF2F1 | general transcription factor IIF subunit 1(GTF2F1) |
| TMPRSS2 | transmembrane protease, serine 2(TMPRSS2) |
| MDM2 | MDM2 proto-oncogene(MDM2) |
| NCOA3 | nuclear receptor coactivator 3(NCOA3) |
| TMF1 | TATA element modulatory factor 1(TMF1) |
| HDAC1 | histone deacetylase 1(HDAC1) |
| SMG5 | SMG5, nonsense mediated mRNA decay factor(SMG5) |
| UBE2I | ubiquitin conjugating enzyme E2 I(UBE2I) |
| NCL | nucleolin(NCL) |
| SOD3 | superoxide dismutase 3, extracellular(SOD3) |
| SOD2 | superoxide dismutase 2, mitochondrial(SOD2) |
| SOD1 | superoxide dismutase 1, soluble(SOD1) |
| RAD52 | RAD52 homolog, DNA repair protein(RAD52) |
| SMARCE1 | SWI/SNF related, matrix associated, actin dependent regulator of chromatin, subfamily e, member 1(SMARCE1) |
| HDAC7 | histone deacetylase 7(HDAC7) |
| UBC | ubiquitin C(UBC) |
| SMARCC1 | SWI/SNF related, matrix associated, actin dependent regulator of chromatin subfamily c member 1(SMARCC1) |
| SMARCA4 | SWI/SNF related, matrix associated, actin dependent regulator of chromatin, subfamily a, member 4(SMARCA4) |
| FKBP5 | FK506 binding protein 5(FKBP5) |
| CCND1 | cyclin D1(CCND1) |
| KLK3 | kallikrein related peptidase 3(KLK3) |
| SIRT1 | sirtuin 1(SIRT1) |
| RANBP9 | RAN binding protein 9(RANBP9) |
| JUN | Jun proto-oncogene, AP-1 transcription factor subunit(JUN) |
| CASP8 | caspase 8(CASP8) |
| TP53 | tumor protein p53(TP53) |
| FHL2 | four and a half LIM domains 2(FHL2) |
| XRCC6 | X-ray repair cross complementing 6(XRCC6) |
| EP300 | E1A binding protein p300(EP300) |
| FOXO1 | forkhead box O1(FOXO1) |
| PEX5 | peroxisomal biogenesis factor 5(PEX5) |
| WRAP53 | WD repeat containing antisense to TP53(WRAP53) |
| XRCC5 | X-ray repair cross complementing 5(XRCC5) |
| CTNNB1 | catenin beta 1(CTNNB1) |
| GAPDH | glyceraldehyde-3-phosphate dehydrogenase(GAPDH) |
| RNF14 | ring finger protein 14(RNF14) |
| NCOA1 | nuclear receptor coactivator 1(NCOA1) |
| UXT | ubiquitously expressed prefoldin like chaperone(UXT) |
| RUVBL2 | RuvB like AAA ATPase 2(RUVBL2) |
| NCOR1 | nuclear receptor corepressor 1(NCOR1) |
| NCOR2 | nuclear receptor corepressor 2(NCOR2) |
| SP1 | Sp1 transcription factor(SP1) |
| ESR1 | estrogen receptor 1(ESR1) |
| PRDX1 | peroxiredoxin 1(PRDX1) |
| NR0B2 | nuclear receptor subfamily 0 group B member 2(NR0B2) |
| XDH | xanthine dehydrogenase(XDH) |
| MAPK8 | mitogen-activated protein kinase 8(MAPK8) |
| SRC | SRC proto-oncogene, non-receptor tyrosine kinase(SRC) |
| HEY1 | hes related family bHLH transcription factor with YRPW motif 1(HEY1) |
| PA2G4 | proliferation-associated 2G4(PA2G4) |
| NR0B1 | nuclear receptor subfamily 0 group B member 1(NR0B1) |
| RCHY1 | ring finger and CHY zinc finger domain containing 1(RCHY1) |
| HNF4A | hepatocyte nuclear factor 4 alpha(HNF4A) |
| FOXA1 | forkhead box A1(FOXA1) |
| AKT1 | AKT serine/threonine kinase 1(AKT1) |
| NCOA2 | nuclear receptor coactivator 2(NCOA2) |
| PAX6 | paired box 6(PAX6) |
| CDK6 | cyclin dependent kinase 6(CDK6) |
| ATM | ATM serine/threonine kinase(ATM) |
| RUVBL1 | RuvB like AAA ATPase 1(RUVBL1) |
| GRSF1 | G-rich RNA sequence binding factor 1(GRSF1) |
| EGFR | epidermal growth factor receptor(EGFR) |
| NKX3-1 | NK3 homeobox 1(NKX3-1) |
| GSK3B | glycogen synthase kinase 3 beta(GSK3B) |
| GSN | gelsolin(GSN) |
| GSS | glutathione synthetase(GSS) |
| GSR | glutathione-disulfide reductase(GSR) |
| KDM1A | lysine demethylase 1A(KDM1A) |
| KAT5 | lysine acetyltransferase 5(KAT5) |
| TEP1 | telomerase associated protein 1(TEP1) |
| SMG6 | SMG6, nonsense mediated mRNA decay factor(SMG6) |
| NONO | non-POU domain containing, octamer-binding(NONO) |
| STAT3 | signal transducer and activator of transcription 3(STAT3) |
| TERF1 | telomeric repeat binding factor 1(TERF1) |
| PELP1 | proline, glutamate and leucine rich protein 1(PELP1) |
| STUB1 | STIP1 homology and U-box containing protein 1(STUB1) |
| KAT2B | lysine acetyltransferase 2B(KAT2B) |
| NR3C1 | nuclear receptor subfamily 3 group C member 1(NR3C1) |
